# Molecular docking approaches in mycetoma: Toward improved patient management

**DOI:** 10.1371/journal.pntd.0013963

**Published:** 2026-02-11

**Authors:** Ali Awadallah Saeed, Marwa Salah S. Osman, Ahmed Hassan Fahal

**Affiliations:** 1 The Mycetoma Research Center, University of Khartoum, Khartoum, Sudan; 2 Department of Pharmacology and Therapeutics, Faculty of Pharmacy, National University, Khartoum, Sudan; 3 Department of Pharmaceutical Chemistry, Karary University, Omdurman, Sudan; Faculdades Pequeno Príncipe: Faculdades Pequeno Principe, BRAZIL

## Abstract

Mycetoma is a neglected tropical disease characterised by chronic, granulomatous inflammation of the subcutaneous tissues, often leading to disfigurement, disability, and significant socioeconomic burdens. Caused by a diverse array of bacterial and fungal pathogens, eumycetoma is predominantly driven by *Madurella mycetomatis*, and current treatment strategies are limited and often ineffective. Conventional antifungal therapies, such as itraconazole, require prolonged administration, frequently combined with surgical interventions, yet cure rates remain suboptimal, and recurrence is common. The formidable protective grain, comprising microbial material, melanin, and host-derived substances, acts as a physical and biochemical barrier, impeding the penetration and efficacy of drugs. Additionally, issues such as toxicity, resistance, and high costs further complicate management, underscoring the urgent need for novel therapeutic strategies. Recent advancements in computational drug discovery, particularly molecular docking, offer promising avenues to accelerate the identification of effective anti-mycetoma agents. Molecular docking simulates the interaction between small molecules and target proteins, enabling rapid virtual screening of large compound libraries, including natural products, existing drugs, and synthetic molecules, against key pathogenic targets. This structure-based approach helps prioritise candidates with high binding affinity, guiding subsequent experimental validation and reducing both time and financial costs associated with traditional drug development. When integrated with artificial intelligence (AI) and machine learning (ML), these methods can enhance predictive accuracy, uncover novel bioactive scaffolds, and facilitate the repurposing of FDA-approved drugs such as montelukast and vilanterol. Key molecular targets in *M. mycetomatis* include enzymes and pathways critical for pathogen survival and virulence, notably cytochrome P450 (CYP51), dihydrofolate reductase (DHFR), chitin synthase, melanin biosynthesis pathways, and metal ion acquisition systems. Melanin production, via DHN-melanin, DOPA-melanin, and pyomelanin pathways, contributes to grain pigmentation and structural integrity, while metal ions such as iron and zinc are vital for enzymatic activities, grain formation, and fungal virulence. Disrupting metal ion homeostasis through targeting zincophores, siderophores, and zinc-binding proteins represents a promising therapeutic strategy to weaken grain robustness and enhance drug penetration. Despite the potential of molecular docking, limitations such as reliance on homology models, static protein structures, and the absence of cellular context necessitate complementary approaches, including molecular dynamics simulations and in vitro validation. These combined efforts can refine candidate compounds, optimise binding affinities, and predict pharmacokinetic properties. Furthermore, integrating docking results with clinical data and global collaboration platforms can accelerate the discovery of affordable, effective treatments tailored to endemic regions. In conclusion, leveraging molecular docking and computational methods to target essential *M. mycetomatis* pathways offers a promising frontier in mycetoma research. By identifying novel inhibitors and understanding pathogen biology at a molecular level, these approaches can inform targeted therapies, reduce treatment durations, and improve patient outcomes. Future research should focus on validating computational predictions experimentally and translating these findings into clinical practice, with an emphasis on accessible, cost-effective interventions for vulnerable populations affected by this neglected disease.

## Introduction

Mycetoma is a chronic, granulomatous inflammatory infection of the subcutaneous tissues that progressively involves the skin, deeper structures, and bones [1]. Clinically, it presents as painless subcutaneous masses, sinus tract formation, and discharges containing characteristic grains [2]. The disease is caused by a variety of microorganisms, including bacteria that lead to actinomycetoma and fungi that cause eumycetoma [3]. Recognised by the World Health Organization as a neglected tropical disease (NTD), mycetoma predominantly affects impoverished communities with limited access to healthcare and low socioeconomic status, sharing common features with other neglected tropical diseases [4]. Its impact extends beyond individual health, causing significant social, economic, and healthcare burdens in endemic regions.

Mycetoma-causing agents are distributed worldwide but are particularly prevalent in tropical and subtropical zones, collectively known as the ‘Mycetoma belt.’ This region encompasses countries such as Chad, Ethiopia, India, Mauritania, Mexico, Senegal, Somalia, Sudan, Thailand, Venezuela, and Yemen. Although reported cases vary by country, Mexico and Sudan currently report the highest numbers [5].

## Limitations of current treatments

Treating eumycetoma with antifungal medications alone often fails to achieve a complete cure [6]. Consequently, medical therapy is frequently combined with surgical procedures at various stages of treatment [7]. Antifungal drugs play a crucial role by helping to localise and enhance granuloma formation, which facilitates surgical removal [8]. Historically, ketoconazole was the first-line treatment in the 1980s at a dose of 200 mg/day; however, its use was discontinued due to severe hepatotoxicity and adrenal suppression concerns raised by the US Food and Drug Administration (FDA) [9,10]. Over recent decades, several antifungal agents have been used, with itraconazole emerging as the preferred choice, achieving cure rates ranging from 35% to 85% [11,12]. More recently, fosravuconazole has shown promise due to its favourable safety profile, ease of administration, and weekly dosing schedule [13,14].

Currently, itraconazole has become the treatment of choice, typically administered at 400 mg/day for six months in cases with moderate to large lesions (5–10 cm or >10 cm in diameter) or bone involvement, often followed by surgical excision [15]. For smaller lesions, wide local excision combined with three months of itraconazole is recommended, with subsequent ultrasound reassessment. Despite this combined approach, cure rates remain suboptimal, with recurrence rates of at least 33% reported [16].

Current treatments face several challenges, including the necessity for prolonged therapy spanning months or years, which can lead to adverse effects such as hepatotoxicity, adrenal insufficiency, and impotence, alongside high costs and limited access in endemic areas [17,18]. These limitations often necessitate surgical interventions, which in severe cases may result in amputation, leading to long-term disability, social stigma, and a decreased quality of life, further exacerbating the socioeconomic impact on affected individuals and their families [19,20]. The emergence of antifungal resistance among some strains compounds these issues, increasing the risk of treatment failure and recurrence [21,22]. Therefore, there is an urgent need to develop novel antifungal agents targeting mycetoma-causative pathogens. The application of computer-aided drug design can significantly expedite this process by reducing both the time and financial costs associated with traditional drug discovery methods [23,24].

## The role of computational methods in accelerating drug discovery

Computational techniques have revolutionised drug discovery by streamlining stages, decreasing costs, and enhancing overall efficiency [24–26]. These methods offer valuable insights into molecular interactions, enable prediction of drug properties, and assess potential toxicity, ultimately leading to the development of safer and more effective therapies [27–29]. Various approaches are in use and include:

### Virtual screening

Rapidly evaluates large chemical libraries to identify potential drug candidates that bind to specific target proteins, significantly cutting down the time and expense associated with traditional high-throughput screening [30].

### Structure-based drug design

Utilises detailed 3D structures of target proteins to design molecules that precisely interact with active sites, thereby increasing the likelihood of discovering potent drugs [31].

### Ligand-based drug design

When the structure of the target protein is unknown, this approach relies on known active molecules to identify new potential candidates based on shared structural features [32].

### Molecular modelling and simulation

Simulates molecular behaviours in solution, predicts interactions with proteins, and optimises drug-like properties to improve efficacy and safety profiles [33].

## Artificial intelligence and machine learning in drug discovery

Artificial Intelligence (AI) and Machine Learning (ML) algorithms can analyse large-scale molecular and biological datasets to uncover patterns and predict key drug properties. This capability accelerates the identification and development of new drug candidates [34].

In research on fungal pathogens such as *Aspergillus fumigatus*, deep learning models have been utilised to examine extensive chemical libraries and forecast new non-azole CYP51 inhibitors with significant antifungal efficacy, illustrating AI's capacity to identify scaffolds beyond conventional azole chemotypes [35]. Machine learning-based quantitative structure-activity relationship (QSAR) models have also been used to improve echinocandin derivatives against fungal β 1,3 glucan synthase. This shows how AI-driven methods can speed up the design of drugs that work against specific fungal infections [36]. These examples show how AI/ML and molecular docking can work together to help find new drugs to treat mycetoma.

### DMET prediction

Computational techniques can forecast a drug candidate’s absorption, distribution, metabolism, excretion, and toxicity (ADMET) profiles. Such predictions facilitate the early detection of potential issues, thereby streamlining the drug discovery process [37].

## Key drug targets for mycetoma treatment

A target is considered druggable if it is part of an essential biological pathway crucial for the pathogen's survival and plays a central role within that pathway. A target meeting both criteria qualifies as druggable [38].

Several molecular targets have been identified as crucial in mycetoma pathogenesis, including cytochrome P450 (CYP450), dihydrofolate reductase (DHFR), and chitin synthase [18,39,40]. However, current treatments targeting these pathways often show limited effectiveness. This is primarily due to the protective grain formed by the causative microorganisms, which consists of microbial material, cement-like substances, melanin, and other host-derived materials [41]. This structural barrier significantly hampers drug penetration, reducing bioavailability and therapeutic efficacy against the fungal pathogens [27,28,42,43]. To improve treatment outcomes, it is essential to understand and disrupt the molecular mechanisms responsible for grain formation, thereby enhancing the delivery of drugs.

Research indicates that several pathways contribute to grain formation, notably

### Melanin biosynthesis pathways

Melanin serves a protective function that directly leads to the failure of antifungal treatment in eumycetoma [44]. Its highly cross-linked, hydrophobic structure forms a dense physical barrier that restricts many antifungal molecules penetration into the grain. Melanin also has a great ability to bind to and sequester drugs, which lowers their free concentration at the surface of fungal cells. Melanin is also a strong antioxidant that gets rid of reactive oxygen species and makes oxidative stress caused by drugs and the host less effective [43]. These mechanisms explain why grains with a lot of melanin are so strong and why inhibiting melanin biosynthesis could make the grain structure weaker and make drugs work better.

*Madurella mycetomatis* produces three distinct types of melanin, which contribute to the characteristic black colouration of grains

#### DHN-melanin.

Produced via the polyketide synthase (PKS) pathway, involving 1,8-dihydroxynaphthalene and downstream reductases/dehydratases.

#### DOPA-melanin.

Formed through the oxidation of L-tyrosine by tyrosinases or laccases.

#### Pyomelanin.

Derived from homogentisate through tyrosine catabolism, dependent on the enzyme HmgA.

In vitro studies confirm the production of DHN and pyomelanin, while in vivo infection models, such as larvae, demonstrate simultaneous synthesis of DOPA and DHN-melanin. These melanin pathways contribute to increased fungal virulence and the structural integrity of the grain [29,30,42,45].

### Metal ion utilisation

The exploitation of metal ion utilisation has emerged as a promising avenue for developing new treatments against *Madurella mycetomatis*. Since essential metal ions such as iron, zinc, and copper are vital for fungal survival, virulence, and enzymatic activities, including cytochrome P450 51 (CYP51), disrupting their homeostasis could serve as an effective antifungal strategy. Notably, iron and zinc play crucial roles in the formation, maturation, and stability of fungal grains, which are hallmark pathological features of mycetoma. Key functions include [46,47]:

**Structural support:** Zinc stabilises enzymes involved in producing the extracellular matrix, contributing to grain integrity.
**Oxidative stress defence:** Zinc activates superoxide dismutase (SOD), protecting fungal cells from reactive oxygen species.
**Melanin production:** Zinc-dependent enzymes, such as laccase, are essential for melanin synthesis, which enhances grain resilience.

Therefore, interfering with the acquisition or regulation of these metal ions may open up novel therapeutic approaches.

Computational studies focussed on melanin biosynthesis and metal-ion acquisition in fungal diseases offer valuable methodological frameworks, while applications to *Madurella mycetomatis* are still limited. For melanin pathways, numerous virtual screening and docking studies have investigated inhibitors of 4-hydroxyphenylpyruvate dioxygenase (HppD), a crucial enzyme in pyomelanin synthesis, including pharmacophore modelling, docking, and molecular dynamics simulations to pinpoint promising scaffolds. These experiments, primarily involving melanogenic fungus like Aspergillus and Cryptococcus, illustrate the viability of computationally targeting melanin biosynthesis. Similarly, docking pipelines have been applied to fungal metalloproteases, including zinc-dependent enzymes that are implicated in tissue invasion and grain maturation. Nonetheless, despite their biological significance in eumycetoma, there have been no published docking or virtual screening studies assessing HppD, laccases, zinc-binding proteins, or metal-acquisition systems in *M. mycetomatis*. This absence of computational analysis signifies a significant research deficiency and underscores the pressing necessity to broaden structure-based drug discovery initiatives beyond CYP51 to other virulence-related pathways.

#### Zincophores or siderophores.

*Madurella (M) mycetomatis* can maintain zinc homeostasis through Zincophores or Siderophores. These are small molecules or siderophore-like chelators secreted by fungi to scavenge zinc (Zn^2^⁺) from the host environment. In fungal infections, metal-ion acquisition systems depend on certain categories of chelators, necessitating precise terminology to prevent conceptual ambiguity. Classical siderophores are specialised molecules that bind ferric iron (Fe^3^⁺) with very high affinity. Zincophores or zinc-binding chelators, on the other hand, help *Madurella mycetomatis* take in zinc (Zn²⁺), which is a metal that helps enzymes stay stable, protects against oxidative stress, and gives grains their shape. Siderophores are often regarded as iron-specific; however, several fungi synthesise siderophore-like compounds that may chelate zinc and operate similarly to zincophores. This has been shown in *Aspergillus fumigatus*, where proteins such Aspf2 help the fungus get zinc from outside the cell. Similar systems may be present in *M. mycetomatis*, although they have not yet been delineated. The amended text employs the phrase “zincophores and siderophore-like chelators” to appropriately convey this distinction, highlighting that zinc absorption in *M. mycetomatis* is presumably facilitated by zinc-specific or zinc-adapted processes [48].

Because zinc is so important for fungal growth, DNA repair, and enzymatic functions (such as the work of metalloproteases and transcription factors), *M. mycetomatis* probably uses zinc-acquisition mechanisms to compete with the host for this important micronutrient. Even while zincophores have not yet been explicitly found in *M. mycetomatis*, data from other fungi indicates the presence of similar zinc-binding mechanisms [48].

#### Zinc-binding proteins.

These likely include:

**Zinc-dependent enzymes**, such as metalloproteases, superoxide dismutases, and alcohol dehydrogenases, play a vital role in fungal metabolism and stress response [49].
**Transcription factors** (e.g., ZafA-like regulators) that modulate zinc homeostasis [50].
**ZRT/IRT-like transporters** (from the ZIP family) are responsible for zinc uptake, especially under zinc-limited conditions [51].

Targeting zinc acquisition by inhibiting zincophores or zinc transporters could impair fungal growth and enhance the efficacy of existing antifungals, such as itraconazole. Chelation strategies or small-molecule inhibitors targeting zinc-binding proteins may represent innovative treatments for mycetoma. Further research is needed to identify and characterise these systems in *M. mycetomatis.*

## Fungal proteases and adhesin proteins

Fungal proteases and adhesins play crucial roles in the degradation of host tissue and the aggregation of pathogenic proteins during grain formation [52]. In *Madurella mycetomatis*, proteases are key virulence factors that facilitate tissue invasion, immune evasion, and nutrient acquisition [33,53].

Various potential proteases have been identified, including

- **Metalloproteases (Zn**^**2**^**⁺-dependent):** Likely involved in breaking down host tissue and remodelling the extracellular matrix (ECM), such as collagenase-like enzymes [54].
- **Serine proteases:** Possibly contribute to immune evasion by degrading host antimicrobial peptides and complement proteins [55].
- **Aspartic proteases:** May assist in nutrient acquisition by degrading host proteins [56]
- **Secreted subtilisin-like proteases:** Found in other fungal pathogens (e.g., *Aspergillus* spp.) and potentially involved in host cell invasion [57].

## Fungal adhesins in *Madurella mycetomatis*

Adhesins are surface proteins that mediate fungal attachment to host cells and ECM components, promoting colonisation and persistent infection. Potential adhesins include:

- **Hydrophobins:** Surface proteins that enhance adhesion to hydrophobic surfaces such as skin and nails [58].
- **GPI-anchored cell wall proteins:** Likely to bind ECM components like fibronectin, laminin, or collagen within host tissues [59].
- **Mannosyltransferases and adhesin-like proteins:** Similar to *Candida* Als proteins or *Aspergillus* AfaA, these facilitate biofilm formation [60].
- **Lectins (e.g., galectins):** May bind host glycans, strengthening fungal adherence [61].

Targeting these proteases and adhesins could be a strategic approach to destabilise the grain structure, improving antimicrobial delivery, such as itraconazole and enhancing fungal eradication. Further research is essential to identify and characterise these virulence factors in *M. mycetomatis*, as they represent promising targets for drug and vaccine development against mycetoma.

A summary of key druggable targets in *M. mycetomatis*, their cellular localisation, functional roles, and current status in molecular docking studies is provided in Table 1.

**Table 1:** Key molecular targets in *Madurella mycetomatis* and status of molecular docking applications.

Target category	Specific target	Location in fungal cell	Function/role in pathogenesis	Molecular docking status	Known/potential inhibitors
**Membrane Enzymes** [35,62,63]	Cytochrome P450 (CYP51)	Cell membrane	Ergosterol biosynthesis; essential for membrane integrity	**Extensively studied** (Homology modelling, docking, MD simulations)	Azoles (itraconazole, fosravuconazole), montelukast, vilanterol
**Cytoplasmic Enzymes** [64]	Dihydrofolate reductase (DHFR)	Cytoplasm	Folate metabolism; DNA synthesis	**Potential target** (Limited docking studies)	Methotrexate analogs (theoretical)
**Cell Wall Synthesis** [65]	Chitin synthase	Cell wall	Cell wall integrity; structural component	**Potential target**	Nikkomycin Z (in other fungi)
**Melanin Biosynthesis**	Polyketide synthase (PKS) [66,67]	Cytoplasm	DHN-melanin production; grain pigmentation & virulence	**Potential target** (Docking studies in other fungi)	–
	Tyrosinase/Laccase [68–70]	Cytoplasm/Cell wall	DOPA-melanin production; oxidative polymerisation	**Potential target**	Kojic acid, tropolone (in other systems)
	HppD (HmgA) [71,72]	Cytoplasm	Pyomelanin production; tyrosine catabolism	**Potential target** (Virtual screening in other fungi)	NTBC-like inhibitors
**Metal Ion Acquisition**	Zincophores/Siderophore-like chelators [73]	Extracellular	Scavenge Zn^2^ ⁺ /Fe^3^ ⁺ ; metal homeostasis	**Emerging target** (No docking studies in *M. mycetomatis*)	Chelators (deferoxamine, EDTA analogs)
	ZIP transporters (ZRT/IRT-like) [51]	Cell membrane	Zinc uptake under low-Zn conditions	**Emerging target**	–
	Zinc-binding proteins (e.g., metalloproteases, SOD) [74]	Cytoplasm	Enzymatic activity; stress response; grain stability	**Emerging target**	Chelator-based inhibitors
**Virulence Factors**	Metalloproteases [75]	Secreted/Extracellular	Tissue degradation; immune evasion	**Potential target**	Batimastat, marimastat (broad-spectrum)
	Serine/Aspartic proteases [76]	Secreted/Extracellular	Host protein degradation; nutrient acquisition	**Potential target**	Pepstatin A, aprotonin
	Adhesins (Hydrophobins, GPI-proteins) [77]	Cell wall	Host attachment; biofilm formation	**Potential target** (No docking studies)	–
**Transcription Regulators**	ZafA-like regulators [78]	Nucleus/Cytoplasm	Zinc homeostasis regulation	**Potential target**	–

## Promising compounds screened against mycetoma agent targets

In a 2018 study aimed to predict the binding efficacy of various azoles and synthetic compounds to the CYP51 target in *Madurella mycetomatis* by constructing a homology model of MmCYP51 using *Aspergillus fumigatus* CYP51 (PDB: 4UYM) as a template, with 66.09% sequence reported [79]. Molecular docking using MOE software revealed that fosravuconazole exhibited strong interactions with the enzyme, forming hydrogen bonds via its phosphate, nitrile, and triazole moieties. A synthetic compound with notable in vitro activity (IC50 = 0.60 µM) also showed high affinity, emphasising the role of the nitrile group in binding. The model demonstrated predictive value, correlating with experimental data, and suggested fosravuconazole as a promising candidate for treating eumycetoma [80].

A literature search revealed that computational approaches, including homology modelling, virtual screening, free energy calculations, and molecular dynamics simulations, have been used to repurpose FDA-approved drugs [81]. From a library of 2,619 compounds, three promising candidates, montelukast, vilanterol, and lidoflazine, were identified, exhibiting strong binding affinity, stable interactions with CYP51, and dynamic stability, which suggests their potential as novel therapeutic options for eumycetoma [82]. Further investigation is warranted to validate their efficacy.

Recently, a novel CYP51 inhibitor derived from microbial natural products has been developed to enhance treatment options for *Madurella mycetomatis* eumycetoma, which shows limited response to current azole drugs [80]. Using computational approaches, including molecular docking, MM-GBSA binding affinity calculations, ADMET analysis, and molecular dynamics (MD) simulations, researchers screened a library of microbial-derived compounds against a homology model of M. mycetomatis CYP51. From 34 initial hits with superior docking scores compared to itraconazole, nine compounds demonstrated strong interactions with the heme group and key active-site residues [81]. Further refinement identified three promising candidates, monacyclinones G, H, and I, with favourable binding stability and drug-like properties in MD simulations [34]. These findings suggest that microbial natural products may be potential leads for developing new CYP51-targeting antifungals against eumycetoma, although experimental validation remains essential.

## Molecular docking in mycetoma research: Advantages and limitations

Many of the docking studies referenced in this review rely on homology models rather than experimentally determined protein structures, including the MmCYP51 model built using a template with 66.09% sequence identity [83]. Although this level of identity provides a reasonable overall fold, it introduces notable uncertainty at the level of the active site, where small deviations in residue orientation or local loop geometry can significantly affect predicted binding interactions and docking scores. Structural models derived from templates below ~70% sequence identity generally require additional refinement, such as loop optimisation or targeted validation of active-site residues, to minimise these inconsistencies. To address the limitations of static homology models, several studies have incorporated molecular dynamics simulations, which allow the structure to relax, correct local artefacts and sample more physiologically relevant conformations of the binding pocket. Integrating MD with docking therefore provides a more reliable framework for evaluating ligand interactions and strengthens the interpretability of computational predictions, particularly for targets like *Madurella mycetomatis* CYP51 where no crystallographic structures are available [84,85].

While molecular docking significantly expedites mycetoma drug discovery by predicting interactions between drugs and targets, its limitations underscore the importance of integrating methods such as molecular dynamics simulations, laboratory assays, and clinical studies for comprehensive therapeutic development [82,86].

The advantages of molecular docking include cost- and time-efficiency, as it reduces the need for expensive and lengthy experimental screening, focussing on the most promising drug candidates. It enables target-specific drug discovery by facilitating the identification of compounds that selectively bind to *Madurella mycetomatis* CYP51 or other critical fungal targets [87,88]. The approach also enables drug repurposing, allowing for the rapid screening of existing FDA-approved drugs, such as montelukast and vilanterol, for potential anti-mycetoma activity, thereby accelerating the development process. Additionally, it provides insights into binding mechanisms, offering a detailed understanding of key protein-ligand interactions such as hydrogen bonding and hydrophobic contacts, which can inform rational drug design. Furthermore, it complements experimental approaches by working synergistically with in vitro and in vivo studies to validate potential inhibitors before laboratory testing [89].

The limitations of molecular docking involve reliance on homology models, as the crystal structure of *M. mycetomatis* CYP51 has not been experimentally determined [34]. Consequently, docking depends on predicted models that may contain inaccuracies. Most docking techniques treat proteins as rigid structures, neglecting their conformational flexibility, which can influence binding interactions. There is also the potential for false positives and negatives, as scoring functions may misestimate binding affinities, leading to incorrect compound rankings. Docking does not account for biological factors such as cellular uptake, metabolism, or toxicity, and thus requires further experimental validation to confirm predictions [90]. Additionally, for understudied fungal proteins, such as zincophores and adhesins, limited structural data diminishes the reliability of docking predictions.

## Future perspectives and clinical implications

Molecular docking presents substantial opportunities to advance mycetoma drug discovery through high-throughput virtual screening (HTVS). By rapidly evaluating large compound libraries, including natural products, FDA-approved drugs, and synthetic molecules, against critical *M. mycetomatis* targets such as CYP51, proteases, and zincophores, this approach accelerates the identification of promising inhibitors. It offers a cost-effective strategy to filter out weak binders early, enabling researchers to concentrate experimental efforts on the most viable candidates. Integrating artificial intelligence and machine learning can further enhance predictive accuracy and facilitate the discovery of novel bioactive scaffolds, thereby streamlining the entire drug development pipeline.

To translate in silico predictions into clinical applications, systematic validation through *in vitro* and i*n vivo* studies is essential. Top computational hits should undergo antifungal susceptibility testing in the laboratory, followed by validation in animal models such as *Galleria mellonella*. Molecular dynamics simulations and free-energy calculations can support structure-activity relationship (SAR) optimisation, guiding chemical modifications to improve potency and minimise toxicity. Additionally, docking can help identify synergistic combinations, such as pairing new inhibitors with existing antifungals like itraconazole, that may enhance treatment efficacy.

Personalised and targeted therapies are particularly promising for endemic regions, where genetic variability among *M. mycetomatis* strains may require strain-specific treatment strategies. Computational modelling can account for mutations in targets like CYP51, enabling the development of tailored therapeutics. Beyond direct antifungal agents, host-directed approaches that interfere with fungal adhesion or immune evasion mechanisms could provide complementary benefits. Advances in portable sequencing technologies could facilitate point-of-care diagnostics, allowing clinicians in resource-limited settings to detect resistance markers and optimise treatment plans.

Translating computational predictions into clinically effective treatments encounters substantial practical and logistical challenges in areas where mycetoma is endemic. Limited access to high-throughput screening facilities, advanced analytical instrumentation, and chemical libraries hampers the experimental validation of promising in silico findings. Additionally, practical difficulties, such procuring chemicals, creating in vivo infection models, and doing thorough preclinical research, are made worse by limitations in infrastructure and finance. In low-resource health systems, regulatory routes for new antifungal drugs may take a long time or be unclear, which could impede their use in clinical settings. It is also important to carefully think about ethical issues, such as making sure that everyone can take part in the experiment and get access to treatments after the trial. To get beyond these problems, it's important to build up local research expertise, encourage international partnerships, and push for open-access data and compound sharing. Portable diagnostic techniques, including point-of-care sequencing and biomarker detection, could help with individualised treatment plans and testing computational results in real-world situations where diseases are common.

Open-access databases and international collaborations are crucial for advancing mycetoma research. Sharing target libraries and structural models on platforms such as ChEMBL and PubChem can prevent redundant efforts and foster innovation. Collaborative initiatives for drug repurposing, leveraging open-source docking data from repositories like ZINC15 and DrugBank, may reveal overlooked candidates. Integrating computational predictions with real-world clinical data, such as treatment outcomes from the Mycetoma Research Center in Sudan, will refine predictive models and inform effective therapeutic strategies.

While computational approaches are powerful tools for accelerating drug discovery, their true potential depends on validation through experimental and clinical studies. Promoting open science and fostering global partnerships will be crucial in translating these insights into affordable, effective therapies for populations affected by mycetoma in endemic areas.

## Conclusion

Mycetoma, particularly eumycetoma caused by *Madurella mycetomatis*, remains a significant therapeutic challenge due to limited drug efficacy, prolonged treatment courses, the protective grains that impede antifungal penetration, and high recurrence rates. Current therapies, like itraconazole, often necessitate surgical intervention and are associated with high recurrence rates, underscoring the pressing need for novel treatment options. Computational methods, including molecular docking, virtual screening, and molecular dynamics, have already identified promising candidates and highlighted key molecular targets, including CYP51, melanin biosynthesis pathways, metal-ion acquisition systems, and virulence-associated enzymes involved in grain formation and persistence.

Even with these improvements, in silico methods still have limited predictive power because they depend on homology models, don't have enough structural data, and don't have enough biological context. To fix these problems, we need to do systematic experimental validation through both in vitro and in vivo studies. The combination of advanced computational methods, especially molecular dynamics refinement and models based on artificial intelligence and machine learning, has the potential to speed up hit identification, improve chemical scaffolds, and efficiently explore new chemical space.

In the end, the best way to turn computer-generated insights into effective, cheap treatments is to use a synergistic framework that combines docking, molecular dynamics, cheminformatics, and AI-driven discovery, with open-access data sharing and global collaboration. To get past the current treatment problems and make things better for patients with mycetoma, it is important to make this integrated approach stronger.
